# Delocalization error poisons the density-functional many-body expansion†

**DOI:** 10.1039/d4sc05955g

**Published:** 2024-10-30

**Authors:** Dustin R. Broderick, John M. Herbert

**Affiliations:** a Department of Chemistry & Biochemistry, The Ohio State University 151 W. Woodruff Ave. Columbus Ohio 43210 USA herbert@chemistry.ohio-state.edu

## Abstract

The many-body expansion is a fragment-based approach to large-scale quantum chemistry that partitions a single monolithic calculation into manageable subsystems. This technique is increasingly being used as a basis for fitting classical force fields to electronic structure data, especially for water and aqueous ions, and for machine learning. Here, we show that the many-body expansion based on semilocal density functional theory affords wild oscillations and runaway error accumulation for ion–water interactions, typified by F^−^(H_2_O)_*N*_ with *N* ≳ 15. We attribute these oscillations to self-interaction error in the density-functional approximation. The effect is minor or negligible in small water clusters, explaining why it has not been noticed previously, but grows to catastrophic proportion in clusters that are only moderately larger. This behavior can be counteracted with hybrid functionals but only if the fraction of exact exchange is ≳50%, whereas modern meta-generalized gradient approximations including ωB97X-V, SCAN, and SCAN0 are insufficient to eliminate divergent behavior. Other mitigation strategies including counterpoise correction, density correction (*i.e.*, exchange–correlation functionals evaluated atop Hartree–Fock densities), and dielectric continuum boundary conditions do little to curtail the problematic oscillations. In contrast, energy-based screening to cull unimportant subsystems can successfully forestall divergent behavior. These results suggest that extreme caution is warranted when the many-body expansion is combined with density functional theory.

## Abstract 2

Self-interaction error leads to runaway error accumulation when density functional theory is used in conjunction with the many-body expansion.

## Graphical abstract

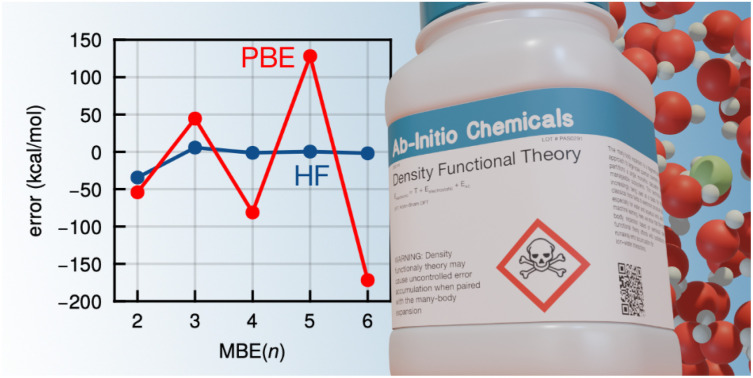

## 1 Introduction

Ostensibly, the many-body expansion (MBE) offers a method-agnostic way to apply electronic structure theory to large molecular systems,^1–4^ avoiding the steep nonlinear scaling of *ab initio* quantum chemistry by partitioning a supersystem into small fragments. The total energy is then approximated as a sum of *n*-body interactions between these fragments:^5^1
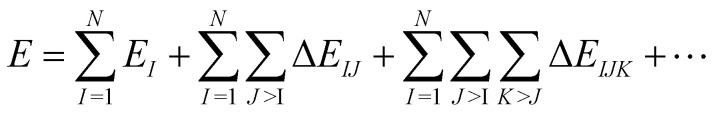
If higher-order terms such as Δ*E*_*IJKL*_ are negligible, then the formal complexity of the electronic structure problem is dramatically reduced. By decomposing a large (and potentially intractable) calculation into a collection of independent or loosely-coupled subsystem calculations, fragment-based quantum chemistry^5–8^ represents one of the most promising ways to extend electronic structure theory to exascale computer architectures.^9^

The MBE in eqn (1) forms the basis of a wide variety of fragment-based approximation schemes.^5–8^ These have been used in hybrid quantum mechanics/molecular mechanics approaches,^10–14^ as a basis for developing classical force fields for water–water and ion–water interactions,^15–31^ as a means to elucidate the nature of intermolecular interactions,^30–38^ and as a way to overcome the dimensionality problem in machine learning.^39–43^ In principle, correlated wave function models can be used for the electronic structure, since only small *n*-body subsystem calculations are required, but density functional theory (DFT) has also been suggested for general use in force-field development.^44^ One can imagine the MBE as a means to accelerate DFT-based *ab initio* molecular dynamics simulations,^45–47^ and the low cost of DFT calculations make this an attractive choice for generating the enormous data sets that are necessary for machine learning applications. It is these DFT-based applications (and potential applications) that concern us.

DFT has become the dominant tool for electronic structure calculations due to its combination of favorable scaling and quantitative or semi-quantitative accuracy for a wide range of chemical problems.^48–50^ Nevertheless, it is not without systemic problems. Among these, perhaps the most pernicious and pervasive is self-interaction error (SIE),^51–54^ also known as delocalization error.^55–57^ Although SIE is most often associated with exaggerated delocalization of unpaired spins,^58–71^ including fractional atomic charges at stretched bond lengths,^51,72–74^ SIE also produces a driving force to delocalize charge in closed-shell cases. It is especially problematic for solvated and condensed-phase ions.^75–80^ In the present work, we demonstrate how delocalization error interacts with the MBE to create a feedback loop leading to runaway error accumulation. The problem is most pronounced for semilocal functionals derived within the generalized gradient approximation (GGA), but is still serious for hybrid functionals such as B3LYP or PBE0, and meta-GGAs such as ωB97X-V^81^ and SCAN.^82^ It significantly impairs the applicability of the DFT-based MBE. That alone should give pause as this method is considered for qualitative analysis, machine learning, or force-field development.

## 2 Results

In what follows, we consider errors in DFT-based MBE(*n*) calculations, meaning that eqn (1) is truncated at *n*-body interactions. Explicit expressions for the *n*-body corrections (Δ*E*_*IJ*_, Δ*E*_*IJK*_, *etc.*) can be found elsewhere.^2^ Calculations were performed with the FRAGME∩T code^83,84^ interfaced to Q-CHEM,^85^ as described in Section 5. For calculations on ion–water clusters X^±^(H_2_O)_*N*_, the property of interest is the ion's interaction energy with the water cluster, Δ*E*_int_. Errors in MBE(*n*) approximations are defined with respect to a counterpoise (CP) corrected,^86^ supramolecular calculation of Δ*E*_int_ at the same level of theory that is used for the *n*-body calculations. The CP correction in the supersystem benchmark is useful for comparison to CP-corrected MBE(*n*) results but it amounts to a constant offset and does not affect the oscillations that are the primary focus of this work.

### 2.1 Fluoride–water clusters

**Fig. 1:**
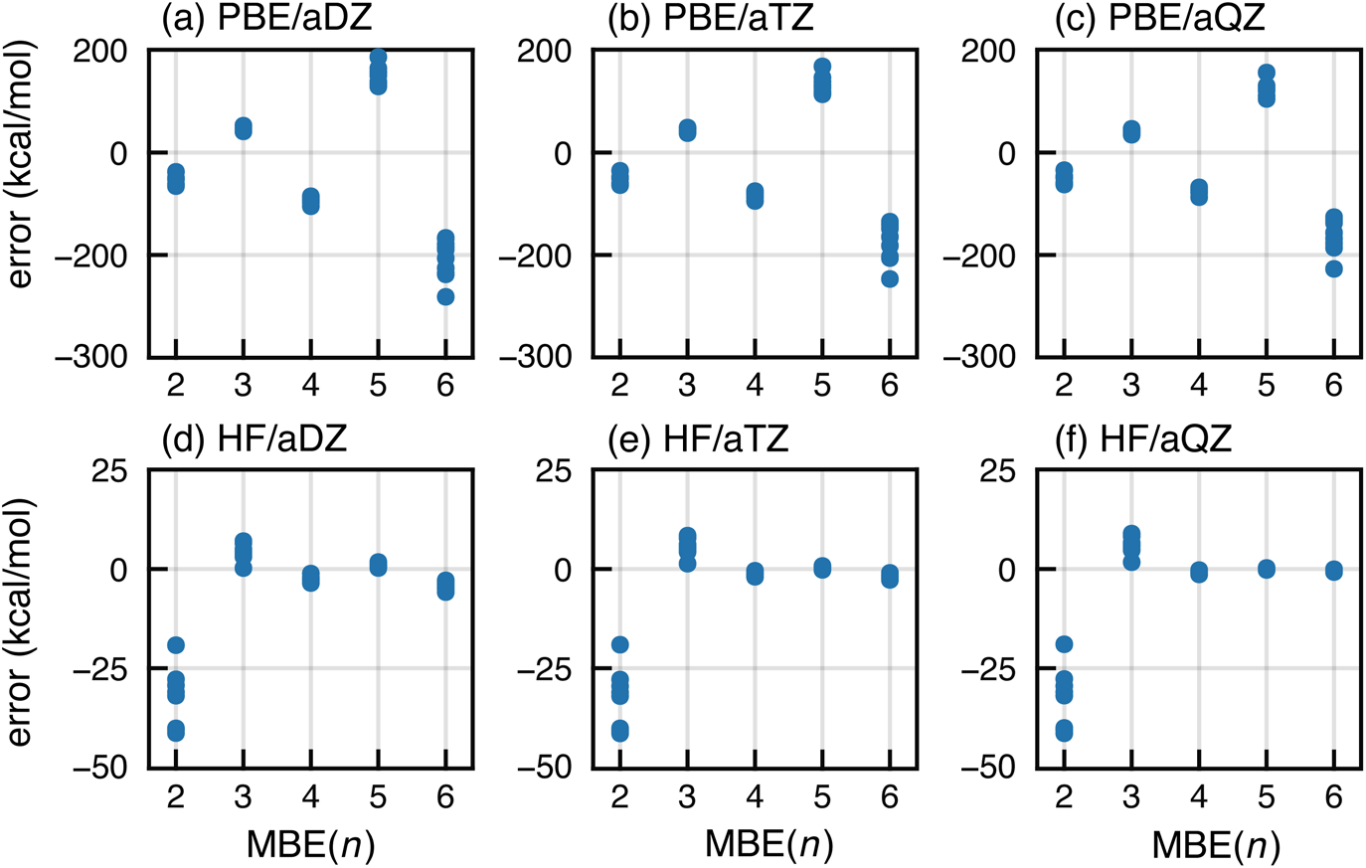
Errors in MBE(*n*) approximations for Δ*E*_int_ in a set of ten F^−^(H_2_O)_15_ clusters, computed at (a)–(c) the PBE/aXZ level (top, for X = D, T, and Q) and (d)–(f) the HF/aXZ level (bottom). In each case, error is defined with respect to a CP-corrected, supramolecular calculation at the indicated level of theory.

We first consider a data set consisting of ten F^−^(H_2_O)_15_ clusters, with calculations performed using either the PBE functional or else Hartree–Fock (HF) theory. We examine basis sets aug-cc-pVXZ (abbreviated “aXZ”) for X = D, T, and Q. Errors in MBE(*n*) approximations up to *n* = 6 are plotted in Fig. 1. Ratios of the MBE(*n*) approximation, Δ*E*_int_[MBE(*n*)], to the CP-corrected supramolecular result (Δ*E*_int_) are plotted in Fig. S1† (analogous ratios are provided in the ESI† for other error plots provided in this work).

The HF-based MBE(*n*) interaction energies converge as expected to the reference supersystem value, with five-body terms that are negligible in what is effectively the basis-set limit, aQZ. Higher-order *n*-body terms can sometimes be artifacts of basis-set superposition error (BSSE),^87,88^ which likely explains the diminished importance of the six-body terms at the HF/aTZ and HF/aQZ levels, relative to HF/aDZ results.

**Fig. 2:**
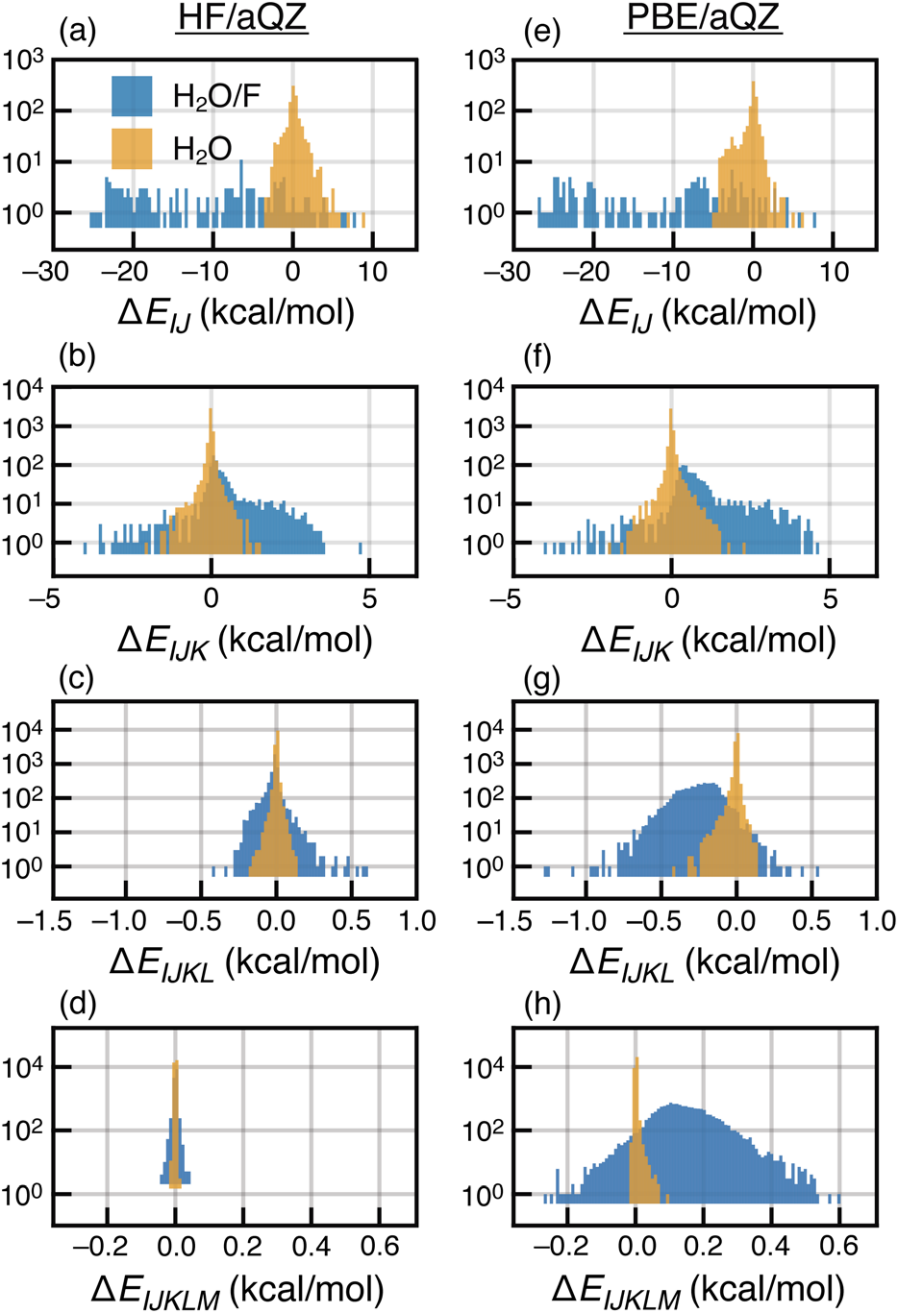
Histograms of all *n*-body interactions Δ*E*_*IJ*⋯_ up to *n* = 5, for MBE(*n*) applied to (a)–(d) F^−^(H_2_O)_15_ at the HF/aQZ level (left) and (e)–(h) the PBE/aQZ level (right). The interaction terms Δ*E*_*IJ*⋯_ that involve F^−^ are shown in blue while those that only involve water are plotted in gold. The overall magnitude of the *n*-body corrections Δ*E*_*IJ*⋯_ decreases with *n* for both methods but for high-order interactions computed using PBE, the fluoride-containing terms are significantly larger than the water-only terms.

In contrast, PBE-based MBE(*n*) calculations are subject to wild oscillations that grow worse as *n* increases; the expansion appears to be divergent for this and other semilocal functionals that we have tested. Histograms of the various interaction terms Δ*E*_*IJ*⋯_ are plotted in Fig. 2 for the HF/aQZ and PBE/aQZ calculations, and summary statistics are provided in Table S1.† The histograms are separated into fluoride-containing subsystems, which afford larger interactions on average, and those that contain only water molecules. Even for the seemingly divergent PBE-based expansions, the magnitude of the *n*-body corrections does decrease with *n*. However, for the PBE calculations the net contribution from the fluoride-containing subsystems *increases* as a function of *n*, leading to the observed divergence. For PBE, the total contribution from the fluoride-containing terms is −115.9 kcal mol^−1^ for *n* = 4 and +193.0 kcal mol^−1^ for *n* = 5. These values are unmatched in the water-only terms, which sum to −4.0 kcal mol^−1^ (*n* = 4) and +1.8 kcal mol^−1^ (*n* = 5). Even those values are still significantly larger than the water-only terms obtained at the HF level, which sum to −0.6 kcal mol^−1^ (*n* = 4) and +0.1 kcal mol^−1^ (*n* = 5).

Thus, the divergent behavior is exacerbated by the presence of an anion. A combinatorial increase in the number of *n*-body terms results in divergence for PBE-based MBE(*n*) despite the fact that individual Δ*E*_*IJ*⋯_ corrections decrease order-by-order. Our data are consistent with previous studies that observed a marked increase in errors for MBE-based energy decomposition analyses (based on two-body terms only) when GGAs were employed.^89,90^ Those studies, however, were limited to (H_2_O)_6_ clusters that do not engender the rapid divergence that we observe using F^−^(H_2_O)_15_.

Four- and five-body terms computed using PBE also show systematic negative and positive biases, respectively, indicating exaggerated magnitude for the higher-order *n*-body corrections. No such systematic error is observed in the HF results, where the *n* = 4 and *n* = 5 histograms are much more symmetric about zero. Assuming oscillating errors in the total energy, consistent with the data in Fig. 1, then for MBE(*n*) to converge it must be the case that2
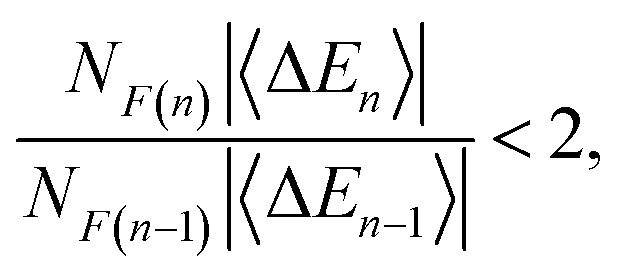
if we assume that 〈Δ*E*_*n*_〉 and 〈Δ*E*_*n*−1_〉 have opposite signs. Here, *N*_*F*(*n*)_ is the number of *n*-body subsystems and 〈Δ*E*_*n*_〉 is the mean *n*-body correction term. Because the number of fragments increases combinatorially, the magnitude of 〈Δ*E*_*n*_〉 can decrease order-by-order (as in Fig. 2) yet the product *N*_*F*(*n*)_〈Δ*E*_*n*_〉 may still be large enough to cause divergence.

**Fig. 3:**
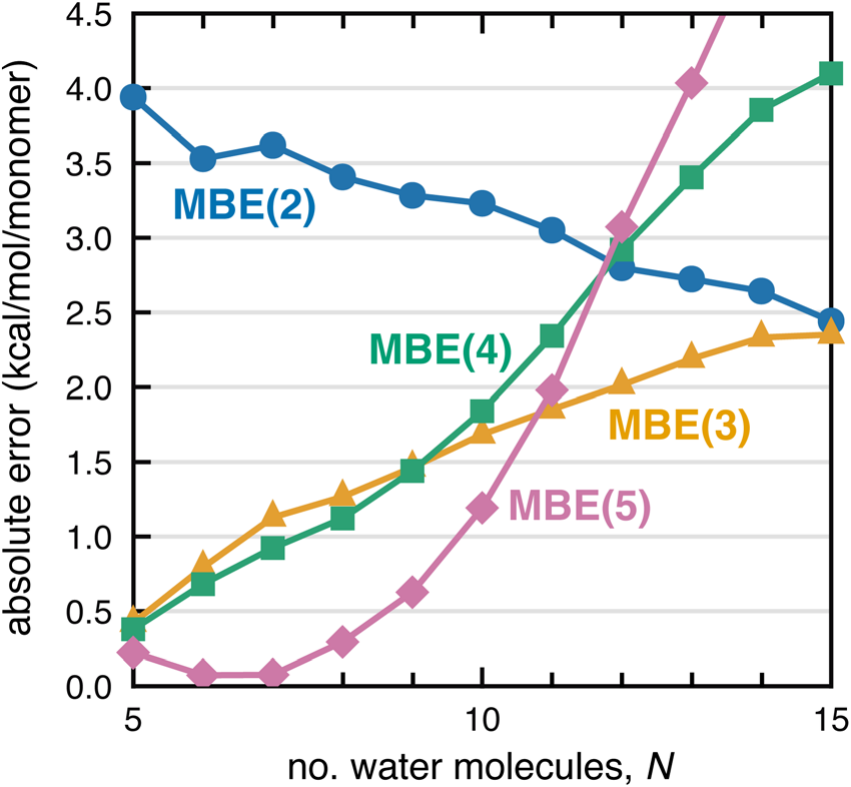
Error per monomer for the F^−^ interaction energy in F^−^(H_2_O)_*N*_ clusters, for calculations at the PBE/aDZ level.

If divergence of MBE(*n*) calculations is indeed driven by combinatorial error accumulation, then smaller clusters with fewer fragments should exhibit improved convergence properties. To examine this hypothesis, we extracted clusters F^−^(H_2_O)_*N*_ with *N* = 5–25 from a molecular dynamics simulation of F^−^(H_2_O)_128_. The absolute error per monomer in Δ*E*_int_, computed at the PBE/aDZ level, is plotted in Fig. 3 up to *N* = 15 and plots up to *N* = 25 can be found in Fig. S5–S7,† for both PBE/aDZ and HF/aDZ calculations. Normalizing the errors by the number of monomers accounts for overall errors that are expected to be size-extensive (*i.e.*, a roughly constant error per hydrogen bond),^91^ and indeed the normalized HF errors in Fig. S5–S7† are independent of cluster size. In contrast, PBE errors diverge as *N* increases, for MBE(3), MBE(4), and MBE(5). We posit that SIE-induced error accumulation explains divergent behavior in DFT-MBE(*n*) calculations that has been documented previously by our group.^1–3^

Examining the PBE-MBE(*n*) results in Fig. 3 and moving up the ladder from *n* = 2 to *n* = 5, we observe an order-by-order reduction in the error for small clusters, up to F^−^(H_2_O)_8_. For larger clusters, however, MBE(4) affords a larger error than MBE(3) and by F^−^(H_2_O)_16_, the two-body expansion affords the smallest error per monomer. For larger clusters, higher-order *n*-body terms are actually detrimental to the accuracy! Considering the product *N*_*F*(*n*)_〈Δ*E*_*n*_〉 in eqn (2) suggests two strategies for improving convergence of MBE(*n*): either reduce the per-fragment error *via* strategies to mitigate SIE, or else reduce the number of fragments *via* screening. Both strategies are pursued in Section 3.

### 2.2 Neutral and cationic clusters

**Fig. 4:**
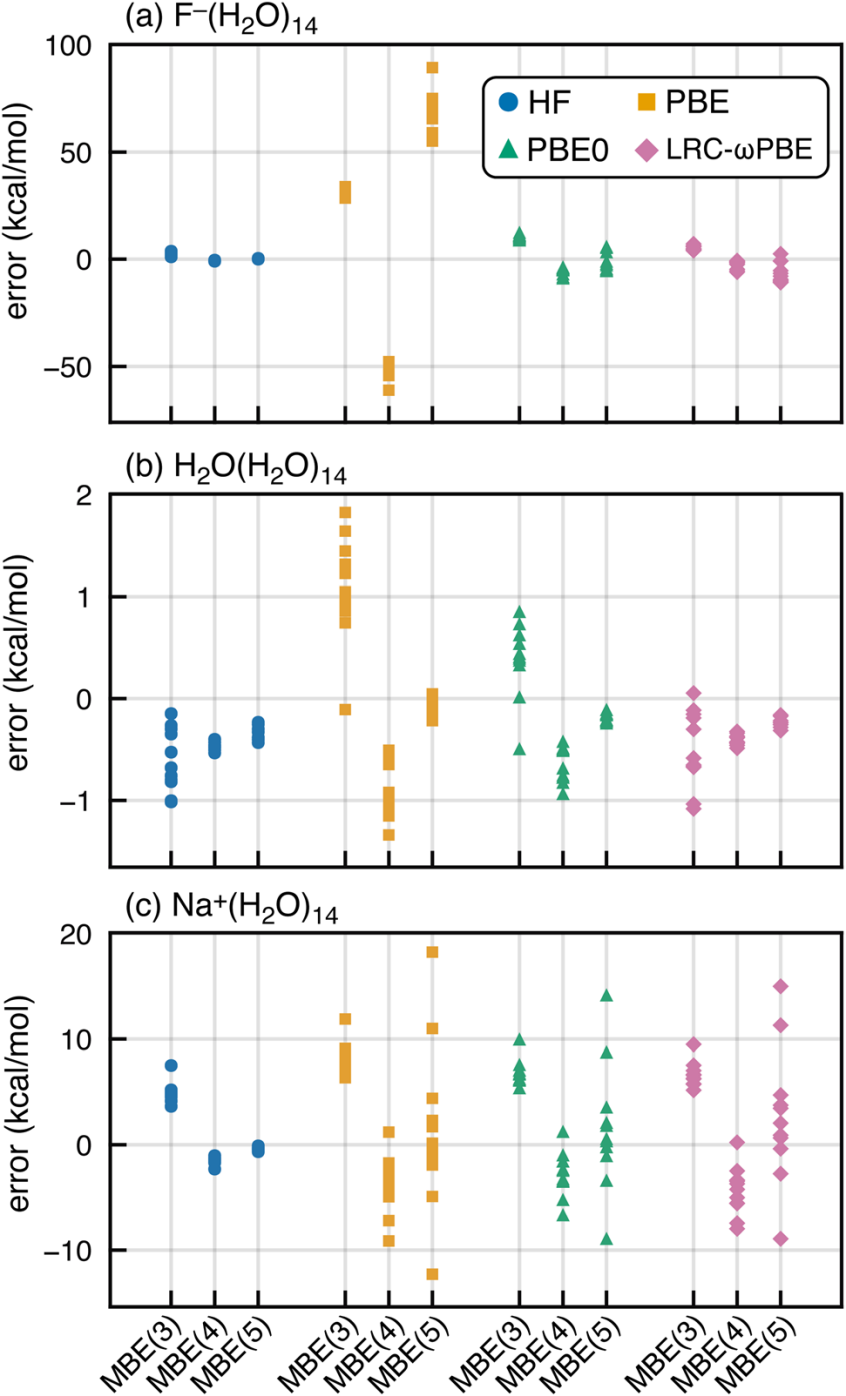
MBE(*n*) errors in Δ*E*_int_ for (a) F^−^(H_2_O)_14_, (b) H_2_O(H_2_O)_14_, and (c) Na^+^(H_2_O)_14_, computed using DFT/aTZ with the functionals indicated. Each data set contains 11 structures extracted from a simulation. In (b), Δ*E*_int_ is defined as the energy to remove a single, central H_2_O molecule whereas in (a) and (c) it is the energy required to remove the ion.

SIE is especially pernicious for anions,^92–100^ so we next consider whether spurious oscillations in MBE(*n*) are limited to hydrated anions. To do so, we extracted a set of (H_2_O)_15_ and Na^+^(H_2_O)_14_ clusters from molecular dynamics simulations. MBE(*n*) results for these systems are plotted in Fig. 4, using the functionals PBE, PBE0, and HF, corresponding to fractions of Hartree–Fock exchange *α*_hfx_ = 0, 0.25, and 1.0, respectively. We also examine results for the long-range corrected LRC-ωPBE functional^101^ that switches between *α*_hfx_ = 0 at short range and *α*_hfx_ = 1 at long range.^101,102^ Results for F^−^(H_2_O)_14_ are also plotted in Fig. 4 to facilitate side-by-side comparison to results from the previous section.

Oscillations in the *n*-body interactions are quite small for the charge-neutral water clusters, albeit still largest with the PBE functional. These oscillations are reduced in magnitude, though not eliminated, by the hybrid functionals. A previous study of hydrated ions using SIE-corrected functionals concluded that SIE is important in F^−^(H_2_O)_*N*_ but not for Na^+^(H_2_O)_*N*_,^103^ although the systems examined were limited to *N* ≤ 2. For Na^+^(H_2_O)_14_, we find that MBE(*n*) diverges using any of the aforementioned functionals except for HF. These exaggerated many-body effects are much larger than what is observed at the three-body level for Na^+^(H_2_O)_2_ or F^−^(H_2_O)_2_,^36,37^ even with semilocal functionals. This difference between microhydrated systems considered in previous studies, and the full solvation-shell clusters examined here, may explain why problems with DFT-based MBE(*n*) calculations have not been reported previously.

In what follows, we will focus on fluoride–water clusters where the problem is most severe but cationic systems are clearly not immune to the SIE problems documented herein. Semilocal functionals also exaggerate many-body effects even in charge-neutral clusters.

## 3 Discussion

### 3.1 SIE exacerbates *n*-body BSSE

A large basis set is vital for minimizing both BSSE and basis-set incompleteness error. Oscillatory convergence of MBE(*n*) towards the supersystem energy is sometimes mitigated in larger basis sets,^104^ but the data in Fig. 1 show little change as the basis set approaches completeness. This suggests that basis-set incompleteness is not primarily responsible for the oscillations that we observe.

**Fig. 5:**
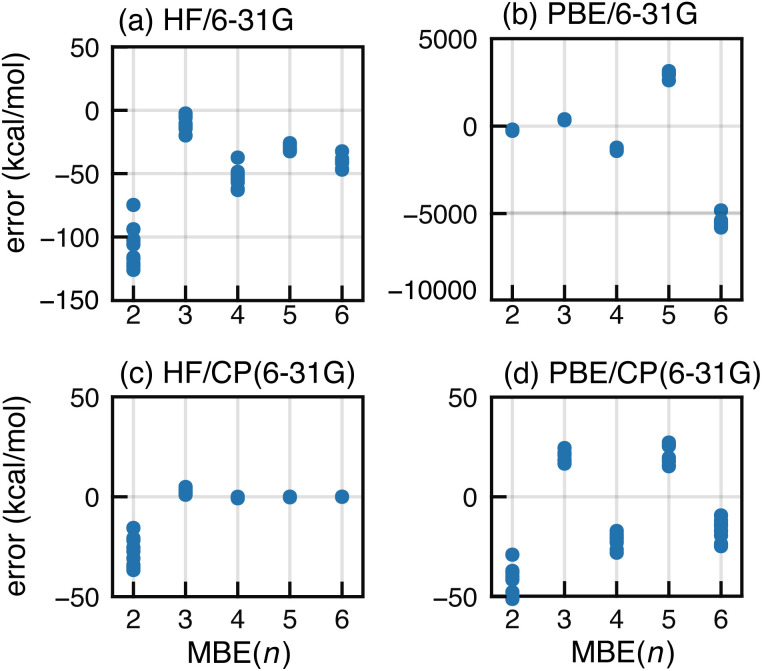
Errors in MBE(*n*) interaction energies for F^−^ in a set of ten F^−^(H_2_O)_15_ clusters at (a) the HF/6-31G level and (b) the PBE/6-31G level. Also shown are results using a full-cluster CP correction, again at the (c) HF/6-31G and (d) PBE/6-31G levels.

BSSE can be eliminated from the MBE(*n*) calculations by performing all subsystem calculations in the full-cluster basis set.^104^ This is somewhat expensive and was pursued using a small basis set, with HF/6-31G and PBE/6-31G results shown in Fig. 5 alongside conventional MBE(*n*) results that use only subsystem basis functions. The latter approach engenders significantly large errors and a −50 kcal mol^−1^ shift in the magnitude of the *n*-body corrections, for both HF and PBE. The CP-corrected HF/6-31G data (Fig. 5c) converge by *n* = 4. For PBE/6-31G, use of the full-cluster basis significantly dampens the magnitude of the oscillations yet they remain quite large, with errors of ∼20 kcal mol^−1^ at the *n* = 3 and *n* = 5 level. The errors that are comparable in magnitude, but opposite in sign, for *n* = 4 and *n* = 6.

The difference between full-cluster HF and PBE results points to the interplay between SIE and BSSE. These effects are coupled because the loss of neighboring basis functions (when the full-cluster basis is replaced by a subsystem basis) confines electron density to a small number of monomers, preventing it from delocalizing throughout space. Similar artificial localization has been observed for anions, where SIE in semilocal functionals leads to an unbound electron that may become artificially bound in a finite basis set.^95–100^ Examples of the same phenomenon include fractional charges on well-separated moieties^72–74^ and charge-sloshing leading to oscillations when MBE(*n*) is applied to proteins with ionic side chains.^105^

As a result of SIE, the mere presence of a distant (therefore, non-interacting) subsystem has a stabilizing effect on the total energy.^56^ In MBE(*n*), both proximate and distant systems are added and removed as *n* changes, resulting in dramatic overstabilization of higher-order *n*-body correction terms when GGA functionals are used. This imbalance is somewhat mitigated by CP correction because the anion's charge can delocalize to other ghost atom sites in each of the subsystem calculations. Absent CP correction, aQZ basis functions extend only about 3 Å beyond the nuclei,^106^ so cannot support a delocalized electron beyond the monomers that are present in the subsystem.

### 3.2 Strategies to reduce SIE

**Fig. 6:**
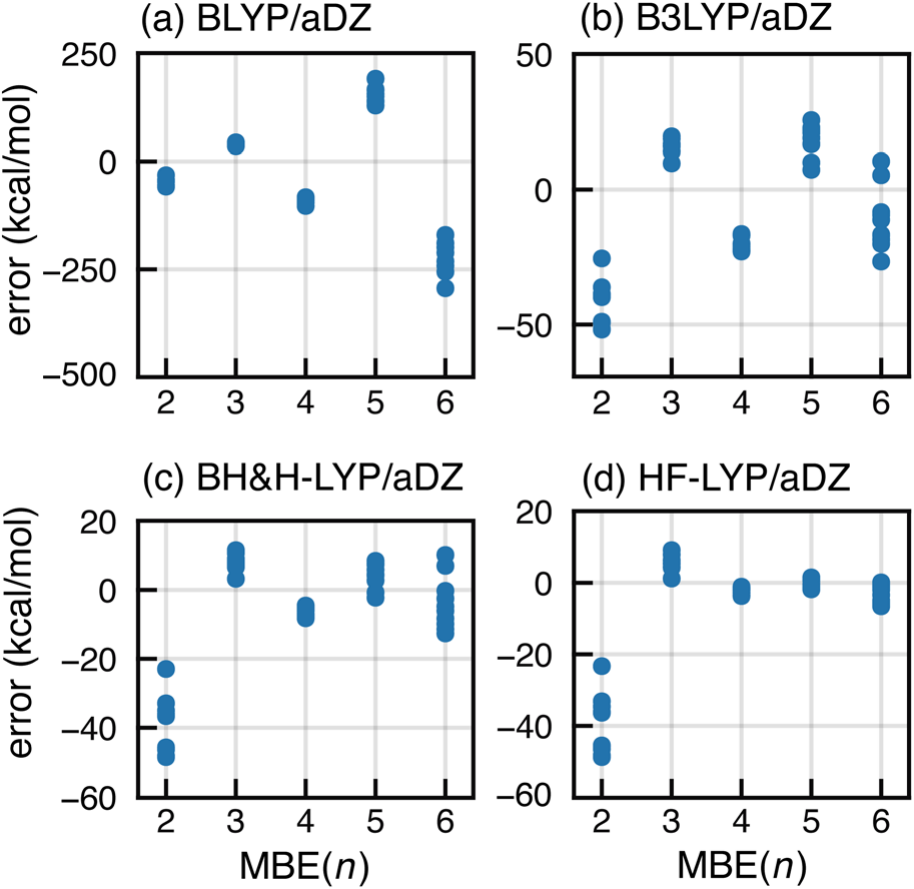
Errors in MBE(*n*) interaction energies for ten configurations of F^−^(H_2_O)_15_ computed using BLYP-based functionals with different fractions of exact exchange: (a) semilocal BLYP functional with *α*_hfx_ = 0, (b) B3LYP hybrid functional with *α*_hfx_ = 0.2, (c) half-and-half functional (BH&H-LYP) with *α*_hfx_ = 0.5, and (d) HF-LYP with *α*_hfx_ = 1. All calculations use the aDZ basis set.

The most common strategy to mitigate SIE or delocalization error is to incorporate a fraction of exact Fock exchange, with a coefficient 0 ≤ *α*_hfx_ ≤ 1. We examine this approach by comparing results for a sequence of functionals: BLYP (with *α*_hfx_ = 0),^107,108^ B3LYP (*α*_hfx_ = 0.2),^108,109^ BH&H-LYP (*α*_hfx_ = 0.5),^110^ and HF-LYP (*α*_hfx_ = 1). As shown in Fig. 6, divergent behavior for F^−^(H_2_O)_15_ persists using B3LYP but results appear to converge for BH&H-LYP, and the oscillations disappear completely for HF-LYP.

Apparently, the BH&H-LYP functional can be used to obtain convergent *n*-body expansions and it is interesting that the same functional often works well in problematic cases of ground- or excited-state charge transfer,^69,70,111–118^ whereas functionals such as B3LYP and PBE0 (the latter with *α*_hfx_ = 0.25) often substantially underestimate charge-transfer energies.^119–124^ In the early days of molecular DFT, the BH&H-LYP functional was assessed as unfit for general-purpose calculations,^72,125^ at least in comparison to B3LYP. Indeed, errors for atomization energies^109,125^ and for reaction energies^126^ are somewhat larger as compared to B3LYP, yet BH&H-LYP is superior to B3LYP for barrier heights.^126,127^ In Table S2,† we compare BH&H-LYP side-by-side with B3LYP (including an empirical dispersion correction for both functionals),^128^ using the GMTKN55 data set.^129^ The overall performance of BH&H-LYP is only marginally worse than that of B3LYP, so the former may be a sensible alternative in cases where standard functionals exhibit SIE problems, including applications of MBE(*n*).

**Fig. 7:**
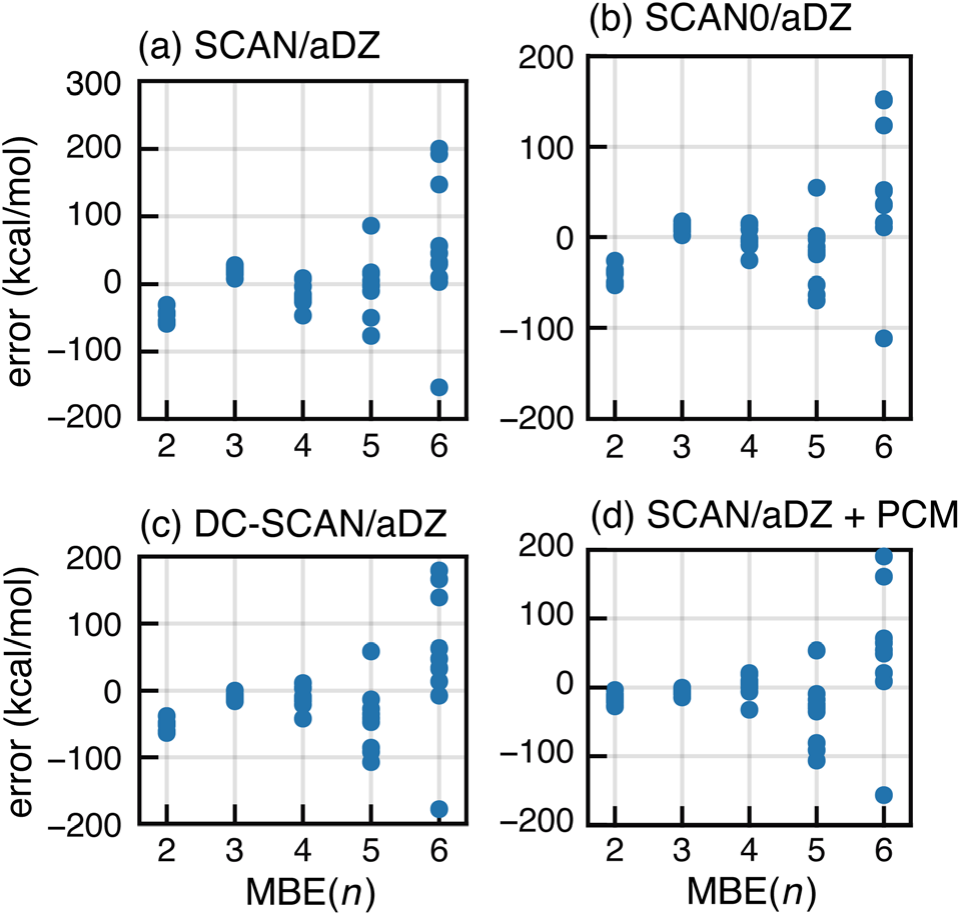
Errors in MBE(*n*) interaction energies for ten configurations of F^−^(H_2_O)_15_, computed using (a) the SCAN functional, (b) SCAN0 (with 25% exact exchange), (c) DC-SCAN, and (d) SCAN in conjunction with dielectric boundary conditions (*ε* = 4). All calculations used the aDZ basis set.

As an alternative to BH&H-LYP, one might consider using newer meta-GGA functionals. Based on tests for (H_2_O)_6_, it has been suggested that the many-body SIE is small for the semilocal SCAN functional.^89^ For F^−^(H_2_O)_15_, however, SCAN exhibits divergent behavior in MBE(*n*) calculations as shown in Fig. 7a. The same is true for the hybrid SCAN0 functional,^130^ which uses *α*_hfx_ = 0.25 (Fig. 7b). We also tried ωB97X-V, which sets *α*_hfx_ = 0.167 for short-range exchange^81^ and is a very good all-around density functional,^50^ but it also exhibits serious oscillations for F^−^(H_2_O)_15_ as shown in Fig. S8b.† Moreover, each of these meta-GGAs is inferior to BH&H-LYP for the SIE-dominated “SIE4 × 4” subset of GMTKN55,^131^ suggesting that these functionals exhibit larger all-around SIE as compared to BH&H-LYP.

Next, we consider the density-corrected^132–135^ (DC-)SCAN approach, also known as “SCAN@HF”,^136^ in which the SCAN exchange–correlation functional is evaluated non-self-consistently atop a self-consistent HF density. This procedure has been shown to reduce density delocalization across hydrogen bonds,^90,136^ and DC-SCAN has been used to generate many-body force fields.^28–30^ In the present calculations, however, DC-SCAN fails to forestall the runaway behavior of MBE(*n*); see Fig. 7c. This observation suggests that promising preliminary results for DC-SCAN applied to small clusters^28–30,136^ do not probe the full extent of problems that are exposed in calculations on a hydrated anion with a complete solvation shell.

Finally, we consider incorporation of low-dielectric boundary conditions as a means to mitigate charge delocalization. In previous work,^105^ we showed that a continuum solvation model with a dielectric constant *ε* = 4 eliminates oscillatory behavior in MBE(*n*) as applied to a large enzyme model with individual amino acids as fragments. The boundary conditions were implemented using a polarizable continuum model (PCM),^137^ not as a model of solvation but rather to reduce charge delocalization that can lead to a vanishing gap and concomitant convergence problems in large-molecule DFT calculations.^138–141^ These problems are sometimes ameliorated by electrostatic stabilization of the molecular surface.^105,140,142^ Notably, convergence is also improved using the BH&H-LYP functional rather than B3LYP or GGAs.^138,139^

MBE(*n*)-SCAN results with boundary conditions corresponding to *ε* = 4 are shown in Fig. 7d but the PCM fails to mitigate the oscillations. As compared to our calculations on proteins,^105^ the fragments used here are rather small and hydrogen bonds may fit within the molecular surface used by the solvent model.^143^ In other words, intermolecular charge delocalization across hydrogen bonds remains possible, and divergent results for MBE(*n*) suggests that this behavior is not mitigated by the low-dielectric PCM.

The same error mitigation strategies that are tested for SCAN in Fig. 7 are examined for PBE in Fig. S5.† The PBE0, DC-PBE, and PBE+PCM methods each significantly reduce (but do not eliminate) oscillatory behavior in MBE(*n*). This suggests that instabilities in MBE(*n*) calculations may manifest differently for GGA *versus* meta-GGA functionals. These functional-dependent differences will be examined in future work.

### 3.3 Screening

High-order MBE calculations quickly become intractable due to combinatorial growth in the number of subsystems, which creates follow-on difficulties for maintaining precision.^1–3^ Therefore, a screening mechanism to reduce the number of subsystems is vital to large-scale deployment of fragmentation, but simple distance-based screening can miss energetically important subsystems.^4,144^ We have shown that energy-based screening is superior in both accuracy and efficiency,^145^ and this type of screening is native to the FRAGME∩T software used here.^83^

We next test the effects of screening on MBE(4) interaction energies for F^−^(H_2_O)_15_, computed at either the HF/aQZ level or the PBE/aQZ level. In either case, three-body fragments are screened using the semi-empirical GFN2-xTB model^146^ with an adjustable threshold *τ*_3_. Four-body subsystems are created from energetically important three-body subsystems, allowing for *M* = 1 missing parents.^83^ This means that tetramer *IJKL* is included if at least three of its four three-body sub-clusters is above threshold (*e.g.*, |Δ*E*_*IJK*_| > *τ*_3_). This procedure has previously been shown to afford energetically converged four-body expansions.^83^

**Fig. 8:**
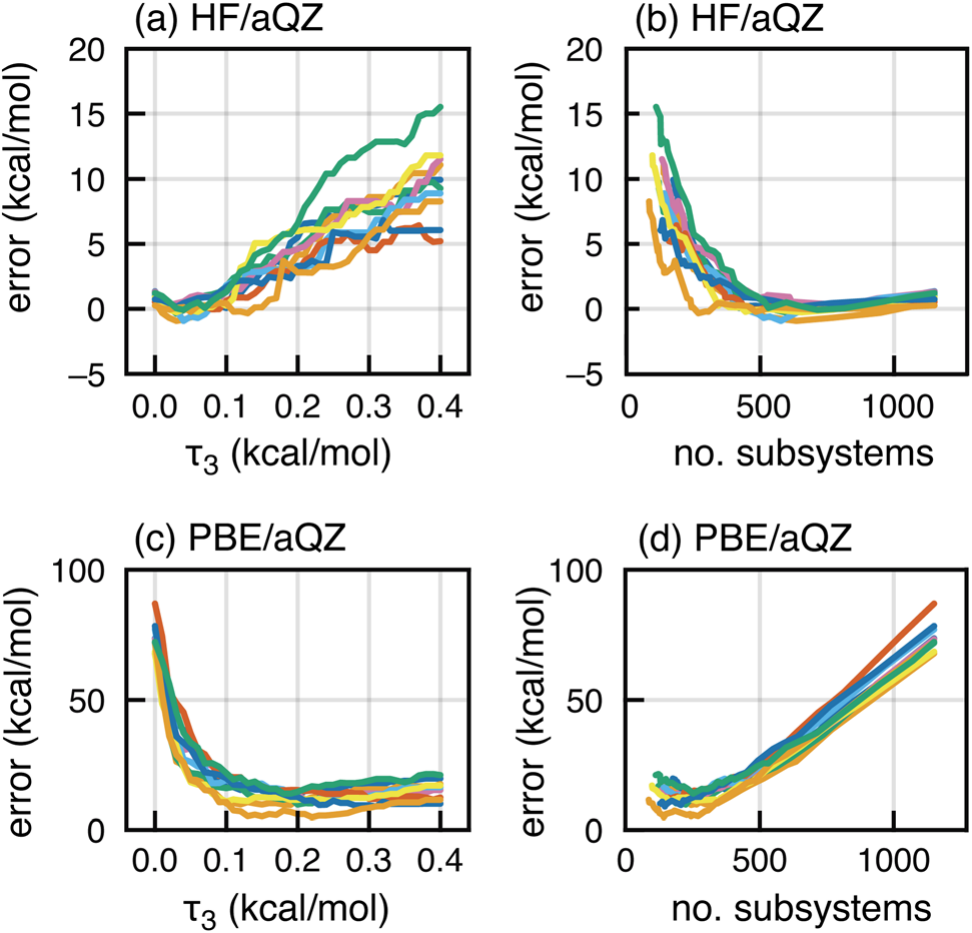
MBE(4) results for ten configurations of F^−^(H_2_O)_15_. (a) Errors in Δ*E*_int_ for calculations at the HF/aQZ level, plotted as a function of the three-body screening threshold *τ*_3_. (b) Same data as in (a), plotted as a function of the number of subsystems. (c) Errors in Δ*E*_int_*versus τ*_3_ for calculations at the PBE/aQZ level. (d) Same data as in (c), *versus* the number of subsystems.

HF/aQZ results in Fig. 8a exhibit minimal error in Δ*E*_int_ when all three-body terms are retained (*τ*_3_ = 0). Errors increase as *τ*_3_ increases and the screening becomes more aggressive, but they remain acceptably small for *τ*_3_ ∼ 0.1 kcal mol^−1^, a value that also affords good results for neutral water clusters.^83^ In Fig. 8b, the same HF/aQZ errors are plotted as a function of the number of subsystems included in the calculation. As expected, errors decrease as the calculation becomes more complete, up to a certain point (around 500 subsystems) when all energetically important terms have been incorporated. Beyond that point there is no further benefit to tightening *τ*_3_, and perhaps some noise introduced as the number of subsystems increases.

These sensible trends are inverted in the PBE/aQZ data, for which the *τ*_3_ = 0 limit engenders catastrophic error accumulation as documented in Section 2.1. Increasing *τ*_3_, which more aggressively removes subsystems from the calculation, dramatically *reduces* the error in Δ*E*_int_; see Fig. 8c. These errors are plotted as a function of the number of subsystems in Fig. 8d. The first *ca.* 250 subsystems do reduce the error, but beyond that the additional subsystems lead to error accumulation. Our interpretation is that a relatively small number of terms is required to get the gross electronic structure correct (meaning that it roughly represents the solvation environment of F^−^ in the full cluster), but once that is achieved any fine details are washed out by cumulative SIE. For large systems, screening not only reduces the cost but also keeps error accumulation in check.

### 3.4 Repercussions and outlook

Paesani and co-workers have suggested DFT-based MBE(*n*) as a tool for generating “data-driven” classical force fields,^44^ and reasonable results have been obtained for neat liquid water and for ion–water interactions using DC-SCAN.^28–30^ In our view, this approach succeeds by limiting the expansion to three-body terms, using a classical polarization model to replace *ab initio* four-body interactions,^29^ and incorporating conservative distance cutoffs.^19–23,30,31^ The generality of this approach is questionable, however, in view of the results presented above. Although four-body terms appear to be sufficient for both neat water^83,88^ and monovalent ion–water interactions,^147^ higher-order interactions are sizable in divalent ion–water clusters.^148^

Our results do suggest there is a “sweet spot” where just enough neighbors are included for accuracy but not so many as to cause significant accumulation of delocalization error; this is exemplified by PBE results in Fig. 8d. However, this may not be sufficient to salvage all ion–water interactions, or for water–solute interactions involving larger, asymmetric solute molecules. A safer strategy is to retreat to HF theory, leveraging the efficiency of the energy-screened MBE to apply post-HF, correlated wave function models. The BH&H-LYP functional so far appears to be a satisfactory workaround with moderate accuracy and DFT cost.

## 4 Conclusions

Pairing MBE(*n*) with DFT results in slow convergence of the *n*-body interactions for neutral systems and rapid divergence for hydrated ions, using a variety of common GGA and meta-GGA functionals. Hybrid functionals with 20–25% Fock exchange also exhibit unphysical oscillations in the *n*-body interactions, and functionals such as BH&H-LYP (with 50% Fock exchange) are required in order to eliminate SIE-induced divergence. The latter functional may be a useful workaround. A DC-DFT correction scheme^132–134^ has shown preliminary promise in small-cluster MBE(*n*) calculations,^28–30,136^ but it does not solve the aforementioned problem in a general way.

These results may have important implications for the application of fragment-based quantum chemistry to study enzymatic reactions, especially for metalloenzymes^149–152^ where different oxidation states of a transition metal might be expected to exhibit varying degrees of SIE. Use of larger fragments may help to mitigate wild oscillations in MBE(*n*) that are documented here, as demonstrated previously for proteins (including those with ionic residues) using low-dielectric boundary conditions.^105^

That strategy is less straightforward in aqueous systems, where the simplest choice is single-H_2_O fragments, although methods with overlapping fragments have been used for calculations on various molecular clusters.^5,153–161^ Screening and culling the subsystem interactions can also mitigate oscillations in MBE(*n*) while simultaneously reducing cost. In that regard, there seems to exist a “Goldilocks point” at which enough solvating water molecules have been included to describe the solute's environment with reasonable accuracy, yet not so many that delocalization error overwhelms the result. Whether this balance can be codified in an unambiguous way remains an important issue for future study.

At present, the safest approach is to rely on MBE(*n*) as an efficient means to apply post-HF correlated wave function methods to large systems, starting from a SIE-free HF calculation and aggressively screening the correlated calculations.^83,145,162–164^ In this way, second-order Møller–Plesset (MP2) calculations have been demonstrated in which a small-basis HF calculation for the entire system is used to recover long-range polarization, with short-range MBE(3) calculations to describe electron correlation, such that the total cost is dramatically reduced with respect to conventional MP2 calculations.^145^ In future work, we will extend this approach to coupled-cluster calculations that can achieve benchmark accuracy for thermochemistry and non-covalent interactions.

## 5 Methods

We have previously reported a “bottom-up” algorithm to implement MBE(*n*) using low-level (typically semi-empirical) energy screening to construct a representation of the *n*-body interactions in the form of a directed acyclic graph.^83^ This is vastly more efficient than order-by-order screening, which allows us to extend the expansion to unprecedented orders (*n*) and systems sizes (*N*).^83,105^ This technique is implemented in an open-source code called FRAGME∩T,^84^ which is used for all of the calculations reported here. Electronic structure calculations are performed by interfacing FRAGME∩T with Q-CHEM.^85^

Interaction energies are computed using the supramolecular approach,

3 Δ*E*_int_(*AB*) = *E*(*AB*) − *E*(*A*) − *E*(*B*).

For noncovalent clusters, MBE(*n*) becomes exact when *n* = *N*. As such, the relevant benchmark for MBE(*n*) is a supersystem calculation using the same functional and basis set. Error is defined as the difference between the MBE(*n*) approximation and the supramolecular benchmark (in the present work, these supramolecular benchmarks are counterpoise corrected). Many-body counterpoise corrections for use with MBE(*n*) have been reported^165–168^ but are not yet implemented in FRAGME∩T.

The MBE(*n*) approximation to Δ*E*_int_ can be computed at a cost that is greatly reduced as compared to naive application of MBE(*n*) to all three terms in eqn (3). This builds on previous work using a generalization of the MBE that can handle overlapping fragments.^169,170^ In this approach, a fragment *F* is simply a subset of the nuclei *A*_*i*_,^83^

4 *F* = {*A*_1_, *A*_2_, …, *A*_*n*_},

and a fragmentation scheme

5 *S*^(*x*)^ = {*F*_1_, *F*_2_, … , *F*_*N*_}

is a collection of fragments, with *x* indicating the current state of the scheme. Scheme *S*^(*x*)^ corresponds to an approximate energy expression6
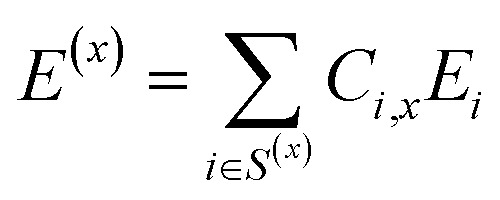
that is a linear combination of subsystem energies *E*_*i*_, with coefficients *C*_*i*,*x*_ derived from the previous state (*x* − 1) using the inclusion/exclusion principle.^83^

To calculate Δ*E*_int_ for a system *F*_*AB*_, we define two subsystems *F*_*A*_ = {*A*_1_, *A*_2_, …} and *F*_*B*_ = {*B*_1_, *B*_2_, …}, such that *F*_*AB*_ = *F*_*A*_ ∪ *F*_*B*_ and *F*_*A*_ ∩ *F*_*B*_ = *∅*. The interaction energy for any fragmentation scheme *S*^(*x*)^_*AB*_ of the system *F*_*AB*_ can be computed as7
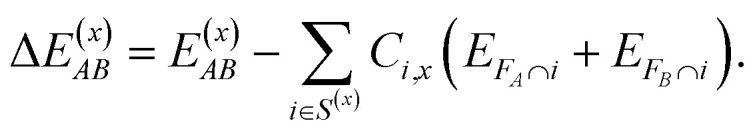
Here, *E*_*F*_*A*_∩*i*_ is the energy of a subsystem *F*_*A*_ ∩ *i* formed from the intersection of *F*_*A*_ with some fragment *i* ∈ *S*^(*x*)^_*AB*_, with a similar meaning for *E*_*F*_*B*_∩*i*_. This is operationally equivalent to dropping all terms in eqn (1) that do not contain nuclei from either *F*_*A*_ or *F*_*B*_. Precomputing coefficients for eqn (7) leads to a substantial reduction in the number of subsystem calculations, simply by avoiding subsystems where *C*_*i*,*x*_ = 0. The number subsystem calculations required to apply MBE(3) to F^−^(H_2_O)_*N*_ clusters ranging up to *N* = 25 is plotted in Fig. S9.† For *N* = 25, *a priori* calculation of the *C*_i,*x*_ coefficients eliminates 78% of the possible subsystems.

In Section 3.3, low-level screening is performed using the GFN2-xTB method,^146^ eliminating terms with

8 |Δ*E*_*IJK*_| < *τ*_3_,

where the threshold *τ*_3_ ranges from *τ*_3_ = 0 (no screening) to *τ*_3_ = 0.4 kcal mol^−1^ (aggressive screening). In building a graph representation of the *n*-body interactions, a new fragment is added to scheme *S*^(*x*)^ if its constituent lower-order terms (“parents”) are present in *S*^(*x*)^. For example, *F*_*ABC*_ is added only if *F*_*AB*_ ∈ *S*^(*x*)^, *F*_*AC*_ ∈ *S*^(*x*)^, and *F*_*BC*_ ∈ *S*^(*x*)^. An additional parameter *M* permits addition of subsystems that are missing at most *M* of their parents. For example, if *M* = 1 then *F*_*ABC*_ is added if two of its three parents {*F*_*AB*_, *F*_*AC*_, *F*_*BC*_} are present in *S*^(*x*)^.^83^

## Data availability

The FRAGME∩T code is available at the URL specified in ref. 84. All molecular structures are provided in the ESI.†

## Author contributions

J. M. H. conceived the project. D. R. B. wrote the code, designed and executed the computational experiments, and performed the analysis. The manuscript was written by both authors.

## Conflicts of interest

J. M. H. is part owner of Q-Chem Inc. and serves on its board of directors.

## Supplementary Material

SC-015-D4SC05955G-s001

SC-015-D4SC05955G-s002
