# Supplementary material for: Delocalization error poisons the density-functional many-body expansion
Source: Chem Sci. 2024 Oct 30;15(47):19893–906. doi: 10.1039/d4sc05955g (PMC11575576; doi:10.1039/d4sc05955g)
Supplement: SC-015-D4SC05955G-s002 [file SC-015-D4SC05955G-s002.pdf]

Supplementary Information (SI) for Chemical Science.
This journal is © The Royal Society of Chemistry 2024

Supplementary Material for:
"Delocalization error poisons the density-functional many-body expansion"

Dustin R. Broderick and John M. Herbert,*
Department of Chemistry & Biochemistry,
The Ohio State University, Columbus, Ohio, 43210 USA

November 6, 2024

S1 Computational Methods

MBE calculations were performed using our FRAGME∩T code,[1] interfaced with the Q-CHEM program[2] for all HF, DFT, and MP2 calculations. The GFN2-xTB method,[3] as implemented in the xTB code,[4] was used for energy screening. For self-consistent field (SCF) calculations in Q-CHEM, the SCF convergence threshold was set to 10^{-8} E_h and the integral screening threshold was set to 10^{-14} a.u.. This should eliminate possible discrepancies between MBE(n) and supersystem calculations for higher-order expansions such as MBE(4).[5] Counterpoise corrections were performed using the usual Boys-Bernardi procedure.[6] For MBE(n) calculations this means that each n-body subsystem calculation is performed using the full-cluster (supersystem) basis set.

S2 Structure Preparation

Structures for $F^-(H_2O)_{15}$ were obtained from a molecular dynamics (MD) trajectory for $F^-(H_2O)_{32}$ performed using GFN2-xTB. This trajectory was propagated for 50 ps using a 2 fs time, with snapshots dumped every 50 fs. The final 10 snapshots were used as the data set for subsequent DFT calculations, taking the 15 water molecules nearest to F^-.

The behavior of MBE(n) as a function of system size was tested using structures obtained from a periodic MD trajectory of $F^-(H_2O)_{128}$, using the AMOEBA force field[7] and the TINKER code.[8] This trajectory was equilibrated for 100 ps and the final snapshot was trimmed to include the N = 5–25 water molecules closest to the ion. An analogous procedure was used to prepare the $(H_2O)(H_2O)_{14}$ and $Na^+(H_2O)_{14}$ structures used in Fig. 4.

Bibliography

[1] FRAGME∩T, https://gitlab.com/fragment-qc/fragment (accessed 2024-10-08).

*herbert@chemistry.ohio-state.edu

[2] E. Epifanovsky, A. T. B. Gilbert, X. Feng, J. Lee, Y. Mao, N. Mardirossian, P. Pokhilko, A. F. White, M. P. Coons, A. L. Dempwolff, Z. Gan, D. Hait, P. R. Horn, L. D. Jacobson, I. Kaliman, J. Kussmann, A. W. Lange, K. U. Lao, D. S. Levine, J. Liu, S. C. McKenzie, A. F. Morrison, K. D. Nanda, F. Plasser, D. R. Rehn, M. L. Vidal, Z.-Q. You, Y. Zhu, B. Alam, B. J. Albrecht, A. Aldossary, E. Alguire, J. H. Andersen, V. Athavale, D. Barton, K. Begam, A. Behn, N. Bellonzi, Y. A. Bernard, E. J. Berquist, H. G. A. Burton, A. Carreras, K. Carter-Fenk, R. Chakraborty, A. D. Chien, K. D. Closser, V. Cofer-Shabica, S. Dasgupta, M. de Wergifosse, J. Deng, M. Diedenhofen, H. Do, S. Ehlert, P.-T. Fang, S. Fatehi, Q. Feng, T. Friedhoff, J. Gayvert, Q. Ge, G. Gidofalvi, M. Goldey, J. Gomes, C. E. González-Espinoza, S. Gulania, A. O. Gunina, M. W. D. Hanson-Heine, P. H. P. Harbach, A. Hauser, M. F. Herbst, M. Hernández Vera, M. Hodecker, Z. C. Holden, S. Houck, X. Huang, K. Hui, B. C. Huynh, M. Ivanov, A. Jász, H. Ji, H. Jiang, B. Kaduk, S. Kähler, K. Khistyaev, J. Kim, G. Kis, P. Klunzinger, Z. Koczor-Benda, J. H. Koh, D. Kosenkov, L. Koulias, T. Kowalczyk, C. M. Krauter, K. Kue, A. Kunitsa, T. Kus, I. Ladjánszki, A. Landau, K. V. Lawler, D. Lefrancois, S. Lehtola, R. R. Li, Y.-P. Li, J. Liang, M. Liebenthal, H.-H. Lin, Y.-S. Lin, F. Liu, K.-Y. Liu, M. Loipersberger, A. Luenser, A. Manjanath, P. Manohar, E. Mansoor, S. F. Manzer, S.-P. Mao, A. V. Marenich, T. Markovich, S. Mason, S. A. Maurer, P. F. McLaughlin, M. F. S. J. Menger, J.-M. Mewes, S. A. Mewes, P. Morgante, J. W. Mullinax, K. J. Oosterbaan, G. Paran, A. C. Paul, S. K. Paul, F. Pavošević, Z. Pei, S. Prager, E. I. Proynov, A. Rák, E. Ramos-Cordoba, B. Rana, A. E. Rask, A. Rettig, R. M. Richard, F. Rob, E. Rossomme, T. Scheele, M. Scheurer, M. Schneider, N. Sergueev, S. M. Sharada, W. Skomorowski, D. W. Small, C. J. Stein, Y.-C. Su, E. J. Sundstrom, Z. Tao, J. Thirman, G. J. Tornai, T. Tsuchimochi, N. M. Tubman, S. P. Veccham, O. Vydrov, J. Wenzel, J. Witte, A. Yamada, K. Yao, S. Yeganeh, S. R. Yost, A. Zech, I. Y. Zhang, X. Zhang, Y. Zhang, D. Zuev, A. Aspuru-Guzik, A. T. Bell, N. A. Besley, K. B. Bravaya, B. R. Brooks, D. Casanova, J.-D. Chai, S. Coriani, C. J. Cramer, G. Cserey, A. E. DePrince III, R. A. DiStasio Jr., A. Dreuw, B. D. Dunietz, T. R. Furlani, W. A. Goddard III, S. Hammes-Schiffer, T. Head-Gordon, W. J. Hehre, C.-P. Hsu, T.-C. Jagau, Y. Jung, A. Klamt, J. Kong, D. S. Lambrecht, W. Liang, N. J. Mayhall, C. W. McCurdy, J. B. Neaton, C. Ochsenfeld, J. A. Parkhill, R. Peverati, V. A. Rassolov, Y. Shao, L. V. Slipchenko, T. Stauch, R. P. Steele, J. E. Subotnik, A. J. W. Thom, A. Tkatchenko, D. G. Truhlar, T. Van Voorhis, T. A. Wesolowski, K. B. Whaley, H. L. Woodcock III, P. M. Zimmerman, S. Faraji, P. M. W. Gill, M. Head-Gordon, J. M. Herbert, and A. I. Krylov, Software for the frontiers of quantum chemistry: An overview of developments in the Q-Chem 5 package, *J. Chem. Phys.*, 2021, **155**, 084801.

[3] C. Bannwarth, S. Ehlert, and S. Grimme, GFN2-xTB—an accurate and broadly parameterized self-consistent tight-binding quantum chemical method with multipole electrostatics and density-dependent dispersion contributions, *J. Chem. Theory Comput.*, 2019, **15**, 1652–1671.

[4] xTB, https://xtb-docs.readthedocs.io/en/latest (accessed 2024-10-08).

[5] K. U. Lao, K.-Y. Liu, R. M. Richard, and J. M. Herbert, Understanding the many-body expansion for large systems. II. Accuracy considerations, *J. Chem. Phys.*, 2016, **144**, 164105.

[6] F. B. van Duijneveldt, J. G. C. M. van Duijneveldt-van de Rijdt, and J. H. van Lenthe, State of the art in counterpoise theory, *Chem. Rev.*, 1994, **94**, 1873–1885.

[7] J. W. Ponder, C. Wu, P. Ren, V. S. Pande, J. D. Chodera, M. J. Schnieders, I. Haque, D. L. Mobley, D. S. Lambrecht, R. A. DiStasio, Jr., M. Head-Gordon, G. N. I. Clark, M. E. Johnson, and T. Head-Gordon, Current status of the AMOEBA polarizable force field, *J. Phys. Chem. B*, 2010, **114**, 2549–2564.

[8] J. A. Rackers, Z. Wang, C. Lu, M. L. Laury, L. Lagardère, M. J. Schnieders, J.-P. Piquemal, P. Ren, and J. W. Ponder, Tinker 8: Software tools for molecular design, *J. Chem. Theory Comput.*, 2018, **14**, 5273–5289.

[9] L. Goerigk, A. Hansen, C. Bauer, S. Ehrlich, A. Najibi, and S. Grimme, A look at the density functional theory zoo with the advanced GMTKN55 database for general main group thermochemistry kinetics, and noncovalent interactions, *Phys. Chem. Chem. Phys.*, 2017, **19**, 32184–32215.

[10] M. Gray, P. E. Bowling, and J. M. Herbert, Comment on "Benchmarking basis sets for density functional theory thermochemistry calculations: Why unpolarized basis sets and the polarized 6-311G family should be avoided", *J. Phys. Chem. A*, 2024, **128**, 7739–7745.

[11] S. Grimme, J. Antony, S. Ehrlich, and H. Krieg, A consistent and accurate *ab initio* parameterization of density functional dispersion correction (DFT-D) for the 94 elements H–Pu, *J. Chem. Phys.*, 2010, **132**, 154104.

List of Figures

S1 Ratio of $\Delta E_{\mathrm{int}}[\mathrm{MBE}(n)]$ (the n-body approximation to ΔE_{int}) and the supramolecular value of ΔE_{int}, for a set of $F^-(H_2O)_{15}$ clusters computed at various levels of theory: (a)–(c) HF/aXZ (for X = D, T, or Q), and (d)–(f) PBE/aXZ. A ratio of unity indicates zero error in the MBE(n) approximation. Errors themselves, computed as $\Delta E_{\mathrm{int}}[\mathrm{MBE}(n)] - \Delta E_{\mathrm{int}}$, are plotted in Fig. 1. . . . . S5

S2 Ratio of $\Delta E_{\mathrm{int}}[\mathrm{MBE}(n)]$ (the n-body approximation to ΔE_{int}) and the supramolecular value of ΔE_{int}, for a set of $F^-(H_2O)_{15}$ clusters computed using (a) HF/6-31G, (b) CP-corrected HF/6-31G, (c) PBE/6-31G, and (d) CP-corrected PBE/6-31G. The corresponding errors are plotted in Fig. 5. . . . . S6

S3 Ratio of $\Delta E_{\mathrm{int}}[\mathrm{MBE}(n)]$ (the n-body approximation to ΔE_{int}) and the supramolecular value of ΔE_{int}, for a set of $F^-(H_2O)_{15}$ clusters computed using (a) BLYP/aDZ, (b) B3LYP/aDZ, (c) BH&H-LYP/aDZ, and (d) HF-LYP/aDZ. The corresponding errors are plotted in Fig. 6. . . . . S6

S4 Ratio of $\Delta E_{\mathrm{int}}[\mathrm{MBE}(n)]$ (the n-body approximation to ΔE_{int}) and the supramolecular value of ΔE_{int}, for a set of $F^-(H_2O)_{15}$ clusters computed using (a) SCAN/aDZ, (b) SCAN0/aDZ, (c) DC-SCAN/aDZ, and (d) SCAN/aDZ augmented with a polarizable continuum model. The corresponding errors are plotted in Fig. 7. . . . . S7

S5 Errors in MBE(n) interaction energies for ten configurations of $F^-(H_2O)_{15}$, computed using (a) the PBE functional, (b) PBE0 (with 25% exact exchange), (c) DC-PBE, and (d) PBE in conjunction with dielectric boundary conditions ($\epsilon = 4$). All calculations used the aDZ basis set. S7

S6 Error in ΔE_{int} for F^-, normalized by the number of monomers, for MBE(3) calculations in $F^-(H_2O)_{5-25}$ clusters at the PBE/aDZ and HF/aDZ levels of theory, plotted as a function of cluster size. S8
S7 Error in ΔE_{int} for F^-, normalized by the number of monomers, for MBE(4) calculations in $F^-(H_2O)_{5-25}$ clusters at the PBE/aDZ and HF/aDZ levels of theory, plotted as a function of cluster size. S8
S8 Error in ΔE_{int} for F^-, normalized by the number of monomers, for MBE(5) calculations in $F^-(H_2O)_{5-25}$ clusters at the PBE/aDZ and HF/aDZ levels of theory, plotted as a function of cluster size. S9
S9 Errors in ΔE_{int} for a set of ten $F^-(H_2O)_{15}$ structures, computed using MBE(n) at various levels of theory: (a) HF/def2-TZVPD, (b) ωB97X-V/def2-TZVPD, (c) RI-MP2/jun-cc-pVTZ, and (d) RI-MP2/aug-cc-pVTZ. These data show that oscillations in MBE(n) terms computed at the RI-MP2 level remain relatively small as compared to those exhibited by density functionals such as ωB97X-V. S9
S10 Total subsystem counts for a complete MBE(3) calculation of ΔE_{int} as compared to the ΔE_{int}-MBE(n) procedure introduced in Section 5. In the former, MBE(3) is applied consistently to all three terms in eqn. (3), whereas the ΔE_{int}-MBE(n) approach involves *a priori* elimination of terms that ultimately cancel. The difference between the two graphs indicates how many fewer subsystems are required in this more efficient approach. S11

List of Tables

S1 Aggregate data for all MBE(n) terms up to $n = 5$, corresponding to the histograms in Fig. 2. All units are given in kcal/mol and standard deviations are given in parentheses. S5
S2 Thermochemical benchmarks for the various subsets of the GMTNK55 data set.[9] Results for BH&H-LYP+D3 are new and results for B3LYP+D3 are taken from Ref. 10. Grimme's D3 dispersion correction[11] is applied to both functionals. S10

Table S1. Aggregate data for all MBE(n) terms up to $n = 5$, corresponding to the histograms in Fig. 2. All units are given in kcal/mol and standard deviations are given in parentheses.

		containing F^-		H_2O only	
	n	mean[a]	total[b]	mean[a]	total[b]
HF/aQZ	2	−10.29 (9.00)	−154.42	0.15 (1.14)	15.75
	3	0.38 (1.01)	40.37	−0.02 (0.17)	−7.94
	4	−0.02 (0.06)	−7.04	0.00 (0.01)	0.63
	5	0.00 (0.00)	0.92	0.00 (0.00)	0.08
PBE/aQZ	2	−11.46 (9.29)	−171.97	−0.29 (1.22)	−30.42
	3	0.90 (1.20)	94.47	0.01 (0.20)	3.40
	4	−0.25 (0.15)	−115.88	−0.00 (0.02)	−3.39
	5	0.14 (0.08)	192.99	0.00 (0.00)	1.76

[a]Mean n-body value based on all possible terms in ten $F^-(H_2O)_{15}$ clusters
[b]Average contribution of n-body terms to a single $F^-(H_2O)_{15}$ cluster.

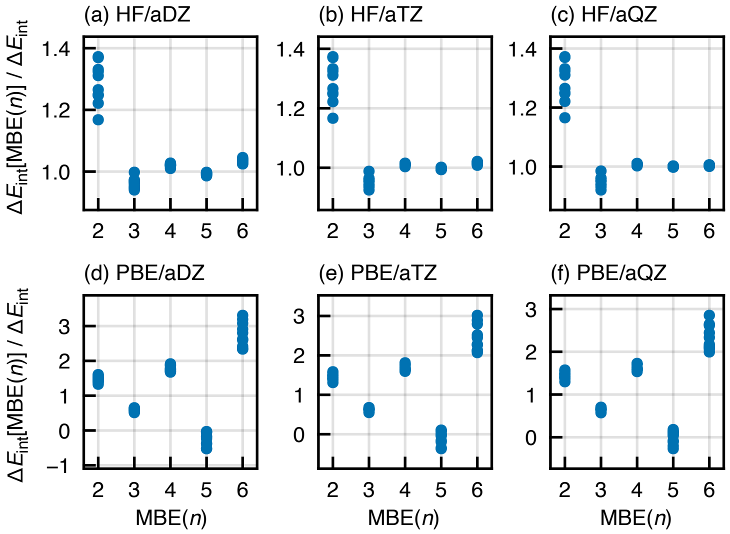

Figure S1. Ratio of ΔE_{int}[MBE(n)] (the n-body approximation to ΔE_{int}) and the supramolecular value of ΔE_{int}, for a set of $F^-(H_2O)_{15}$ clusters computed at various levels of theory: (a)–(c) HF/aXZ (for X = D, T, or Q), and (d)–(f) PBE/aXZ. A ratio of unity indicates zero error in the MBE(n) approximation. Errors themselves, computed as $\Delta E_{\text{int}}[\text{MBE}(n)] - \Delta E_{\text{int}}$, are plotted in Fig. 1.

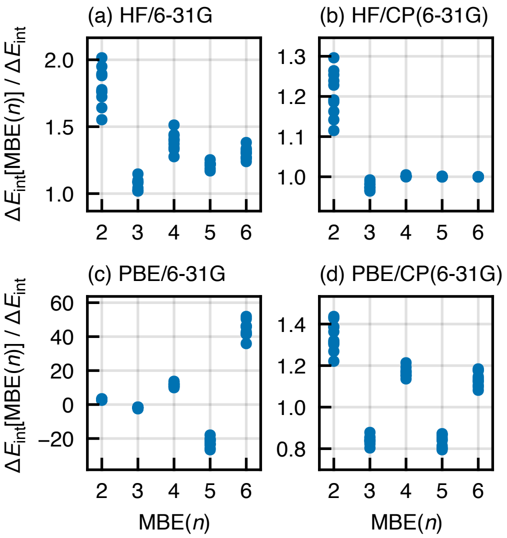

Figure S2. Ratio of $\Delta E_{\text{int}}[\text{MBE}(n)]$ (the n-body approximation to ΔE_{int}) and the supramolecular value of ΔE_{int}, for a set of $F^-(H_2O)_{15}$ clusters computed using (a) HF/6-31G, (b) CP-corrected HF/6-31G, (c) PBE/6-31G, and (d) CP-corrected PBE/6-31G. The corresponding errors are plotted in Fig. 5.

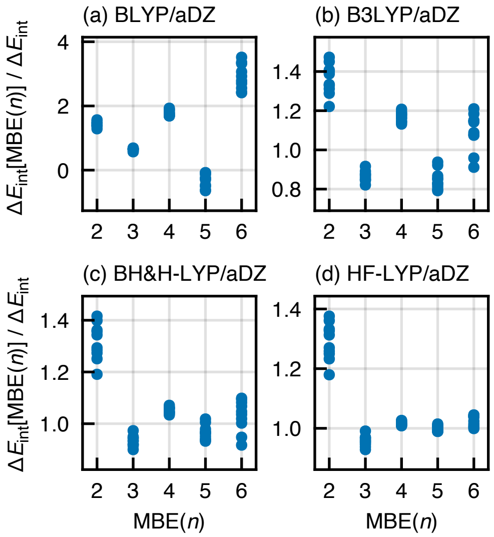

Figure S3. Ratio of $\Delta E_{\text{int}}[\text{MBE}(n)]$ (the n-body approximation to ΔE_{int}) and the supramolecular value of ΔE_{int}, for a set of $F^-(H_2O)_{15}$ clusters computed using (a) BLYP/aDZ, (b) B3LYP/aDZ, (c) BH&H-LYP/aDZ, and (d) HF-LYP/aDZ. The corresponding errors are plotted in Fig. 6.

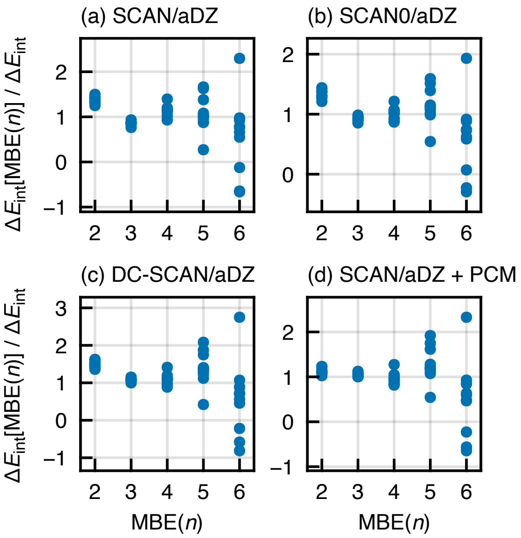

Figure S4. Ratio of $\Delta E_{\rm int}$[MBE(n)] (the n-body approximation to $\Delta E_{\rm int}$) and the supramolecular value of $\Delta E_{\rm int}$, for a set of $F^-(H_2O)_{15}$ clusters computed using (a) SCAN/aDZ, (b) SCAN0/aDZ, (c) DC-SCAN/aDZ, and (d) SCAN/aDZ augmented with a polarizable continuum model. The corresponding errors are plotted in Fig. 7.

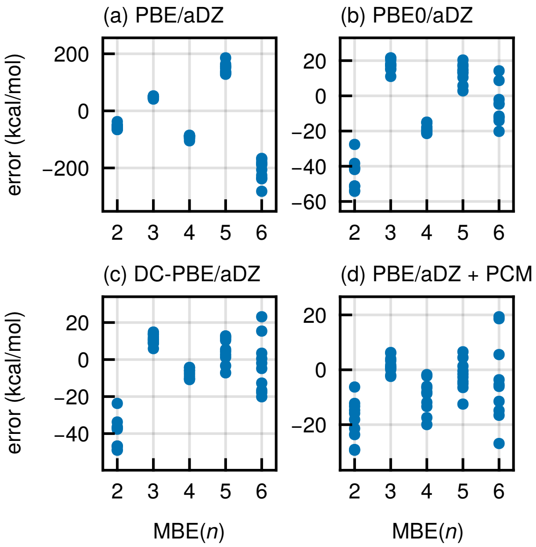

Figure S5. Errors in MBE(n) interaction energies for ten configurations of $F^-(H_2O)_{15}$, computed using (a) the PBE functional, (b) PBE0 (with 25% exact exchange), (c) DC-PBE, and (d) PBE in conjunction with dielectric boundary conditions (ϵ = 4). All calculations used the aDZ basis set.

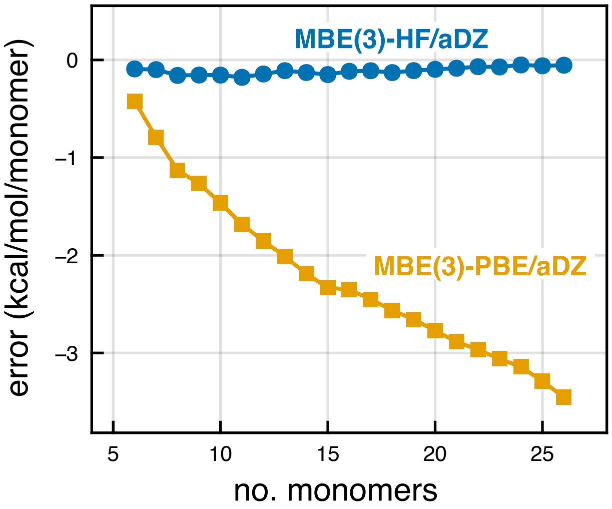

Figure S6. Error in $\Delta E_{\rm int}$ for F^-, normalized by the number of monomers, for MBE(3) calculations in $F^-(H_2O)_{5-25}$ clusters at the PBE/aDZ and HF/aDZ levels of theory, plotted as a function of cluster size.

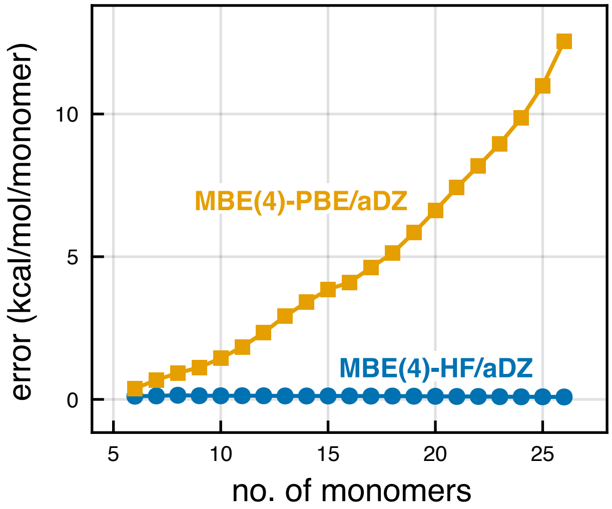

Figure S7. Error in $\Delta E_{\rm int}$ for F^-, normalized by the number of monomers, for MBE(4) calculations in $F^-(H_2O)_{5-25}$ clusters at the PBE/aDZ and HF/aDZ levels of theory, plotted as a function of cluster size.

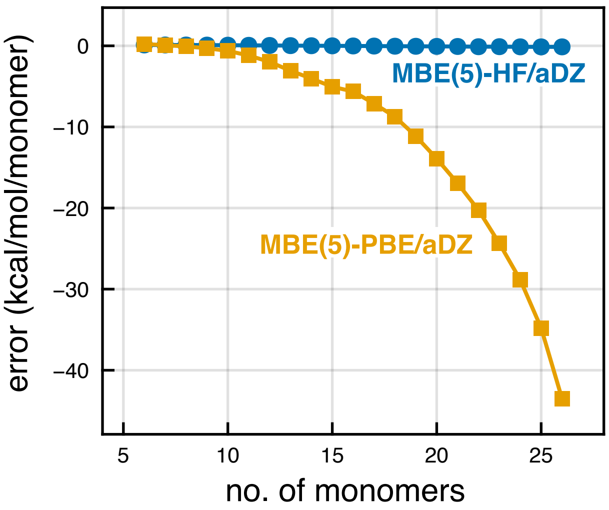

Figure S8. Error in ΔE_{int} for F^-, normalized by the number of monomers, for MBE(5) calculations in $F^-(H_2O)_{5-25}$ clusters at the PBE/aDZ and HF/aDZ levels of theory, plotted as a function of cluster size.

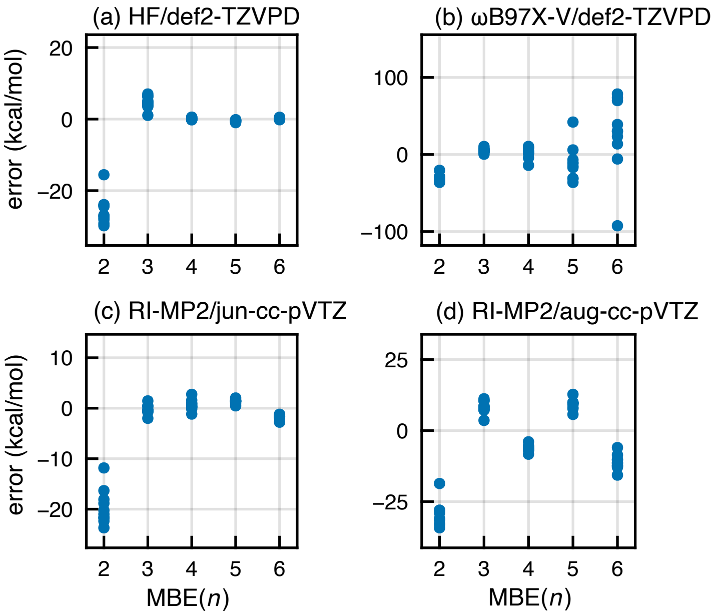

Figure S9. Errors in ΔE_{int} for a set of ten $F^-(H_2O)_{15}$ structures, computed using MBE(n) at various levels of theory: (a) HF/def2-TZVPD, (b) ωB97X-V/def2-TZVPD, (c) RI-MP2/jun-cc-pVTZ, and (d) RI-MP2/aug-cc-pVTZ. These data show that oscillations in MBE(n) terms computed at the RI-MP2 level remain relatively small as compared to those exhibited by density functionals such as ωB97X-V.

Table S2. Thermochemical benchmarks for the various subsets of the GMTNK55 data set.[9] Results for BH&H-LYP+D3 are new and results for B3LYP+D3 are taken from Ref. 10. Grimme's D3 dispersion correction[11] is applied to both functionals.

Data Set	Errors (kcal/mol)									
	BH&H-LYP+D3 / def2-TZVPD					B3LYP+D3 / def2-TZVPD				
	MSE[a]	MAE[b]	STDEV[c]	Max[d]	Min[e]	MSE[a]	MAE[b]	STDEV[c]	Max[d]	Min[e]
FH51	−1.03	1.98	2.43	7.07	0.01	1.63	2.77	3.31	9.13	0.07
INV24	1.35	2.40	3.74	12.60	0.01	−0.74	1.00	1.07	3.49	0.08
BHPERI	4.09	4.09	1.15	6.28	1.47	−0.39	0.98	1.29	3.99	0.02
TAUT15	−0.25	0.55	0.77	2.48	0.01	−0.46	1.14	1.26	2.13	0.10
WCPT18	3.19	3.19	2.40	9.07	0.37	−1.99	2.15	1.91	5.64	0.01
W4-11	−20.95	21.00	13.78	62.62	0.05	−1.97	3.67	5.06	27.59	0.02
PArel	0.51	1.03	1.42	4.20	0.00	−0.04	1.09	1.63	5.14	0.01
DARC	−2.74	5.46	7.00	13.70	0.03	3.63	7.84	7.14	9.81	3.53
PX13	0.95	1.49	1.48	3.11	0.27	−4.99	4.99	1.61	8.01	2.31
G21EA	−6.67	7.49	4.96	13.73	1.16	−0.48	3.25	3.99	10.61	0.04
BSR36	−4.57	4.57	2.53	12.15	1.58	−3.68	3.68	1.99	9.69	1.43
MB16-43	−31.52	34.33	29.90	120.83	3.19	−34.12	34.46	19.01	85.51	0.86
DC13	−0.13	9.68	11.83	24.60	0.03	3.46	10.38	12.75	27.28	0.52
G2RC	−2.90	4.17	4.53	12.18	0.22	1.17	2.79	3.32	8.35	0.00
ISOL24	−0.49	3.54	4.41	10.14	0.06	−2.79	6.03	7.94	23.00	0.01
NBPRC	0.83	2.37	2.61	4.74	0.03	1.70	2.42	3.28	9.94	0.16
G21IP	−1.44	4.50	5.40	12.02	0.08	2.08	3.76	4.40	11.99	0.12
RSE43	−0.30	0.51	0.57	1.35	0.02	−1.74	1.74	0.98	4.49	0.59
DIPCS10	−3.89	7.17	8.00	18.63	1.77	1.78	4.41	5.41	12.60	0.28
YBDE18	−5.73	5.73	2.00	10.62	2.96	−5.37	5.65	4.00	12.32	0.55
BH76	0.05	2.17	2.82	9.90	0.02	−4.89	4.94	2.83	11.16	0.47
SIE4x4	7.14	7.14	6.26	22.30	0.46	17.66	17.66	9.99	40.19	2.80
BH76RC	−0.84	3.72	4.69	12.19	0.13	0.03	2.01	2.58	6.46	0.01
AL2X6	−1.21	1.40	1.06	2.22	0.12	−4.23	4.23	0.73	5.38	3.35
CDIE20	0.53	0.66	0.69	1.94	0.02	1.10	1.12	0.57	2.11	0.20
PA26	2.99	3.25	2.74	9.66	0.12	1.93	2.24	2.47	8.63	0.13
ISO34	0.22	1.26	2.05	9.37	0.00	−0.30	1.88	2.75	10.73	0.02
BHDIV10	2.68	2.68	1.92	5.65	0.15	−1.63	2.88	3.05	5.39	0.18
ALK8	1.70	4.37	5.31	9.97	0.05	−1.09	2.29	2.55	5.64	0.73
BHROT27	0.60	0.60	0.61	1.77	0.02	0.36	0.38	0.43	1.26	0.01
Overall[f]	−1.93	5.08	4.64	14.90	0.48	−1.15	4.79	3.98	12.92	0.62

[a]Mean signed error. [b]Mean absolute error. [c]Standard deviation. [d]Maximum error. [e]Minimum error.
[f]Average across all subsets.

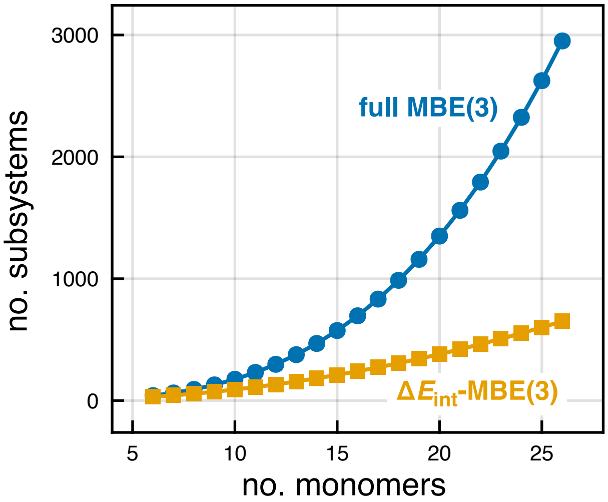

Figure S10. Total subsystem counts for a complete MBE(3) calculation of ΔE_{int} as compared to the ΔE_{int}-MBE(n) procedure introduced in Section 5. In the former, MBE(3) is applied consistently to all three terms in eqn. (3), whereas the ΔE_{int}-MBE(n) approach involves *a priori* elimination of terms that ultimately cancel. The difference between the two graphs indicates how many fewer subsystems are required in this more efficient approach.
